# *catena*-Poly[(diaqua­strontium)-bis­(μ-2-methyl-3,5-dinitro­benzoato)]

**DOI:** 10.1107/S1600536811033769

**Published:** 2011-08-27

**Authors:** Muhammad Danish, M. Nawaz Tahir, Nazir Ahmad, Mehwish Nisa, Iram Saleem

**Affiliations:** a Department of Chemistry, University of Gujrat, Hafiz Hayat Campus, Gujrat, Pakistan; b Department of Physics, University of Sargodha, Sargodha, Pakistan; c Department of Chemistry, University of Sargodha, Sargodha, Pakistan

## Abstract

The title compound, [Sr(C_8_H_5_N_2_O_6_)_2_(H_2_O)_2_]_*n*_, essentially consists of a one-dimensional polymeric network with Sr_2_O_2_ rings extending along the [100] direction. The range of Sr—O bond lengths is 2.4822 (13)–2.8113 (13) Å. C—H⋯O and O—H⋯O hydrogen-bonding inter­actions stabilize the mol­ecules in the form of a two-dimensional polymeric network parallel to (001). One of the nitro groups is disordered over three sets of sites with the occupancy ratio of 0.46:0.32:0.22.

## Related literature

For background information and a related crystal structure, see: Danish, Ghafoor, Ahmad *et al.* (2011*a* ▶,*b* ▶); Danish, Ghafoor, Tahir *et al.* (2011 ▶); Danish, Tahir *et al.* (2011 ▶); Hundal *et al.* (2004 ▶).
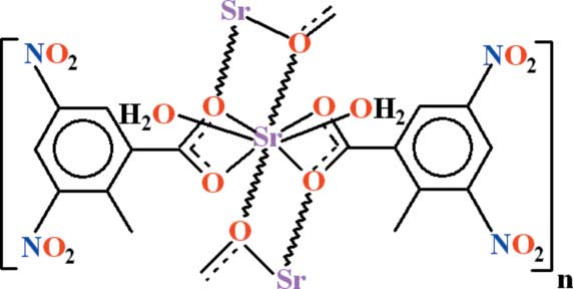


## Experimental

### 

#### Crystal data

[Sr(C_8_H_5_N_2_O_6_)_2_(H_2_O)_2_]
*M* *_r_* = 573.93
Triclinic, 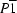

*a* = 8.0901 (3) Å
*b* = 11.2278 (4) Å
*c* = 12.1356 (4) Å
α = 93.805 (2)°
β = 104.566 (1)°
γ = 98.971 (1)°
*V* = 1047.40 (6) Å^3^
*Z* = 2
Mo *K*α radiation
μ = 2.66 mm^−1^
*T* = 296 K
0.30 × 0.26 × 0.22 mm

#### Data collection

Bruker KAPPA APEXII CCD diffractometer
Absorption correction: multi-scan (*SADABS*; Bruker, 2005 ▶) *T*_min_ = 0.457, *T*_max_ = 0.555
15464 measured reflections
3772 independent reflections
3510 reflections with *I* > 2σ(*I*)
*R*_int_ = 0.025

#### Refinement

*R*[*F* ^2^ > 2σ(*F* ^2^)] = 0.023
*wR*(*F* ^2^) = 0.057
*S* = 1.08
3772 reflections
324 parameters
6 restraints
H-atom parameters constrained
Δρ_max_ = 0.41 e Å^−3^
Δρ_min_ = −0.33 e Å^−3^

### 

Data collection: *APEX2* (Bruker, 2009 ▶); cell refinement: *SAINT* (Bruker, 2009 ▶); data reduction: *SAINT*; program(s) used to solve structure: *SHELXS97* (Sheldrick, 2008 ▶); program(s) used to refine structure: *SHELXL97* (Sheldrick, 2008 ▶); molecular graphics: *ORTEP-3 for Windows* (Farrugia, 1997 ▶) and *PLATON* (Spek, 2009 ▶); software used to prepare material for publication: *WinGX* (Farrugia, 1999 ▶) and *PLATON*.

## Supplementary Material

Crystal structure: contains datablock(s) global, I. DOI: 10.1107/S1600536811033769/dn2710sup1.cif

Structure factors: contains datablock(s) I. DOI: 10.1107/S1600536811033769/dn2710Isup2.hkl

Additional supplementary materials:  crystallographic information; 3D view; checkCIF report

## Figures and Tables

**Table 1:** Hydrogen-bond geometry (Å, °)

*D*—H⋯*A*	*D*—H	H⋯*A*	*D*⋯*A*	*D*—H⋯*A*
O13—H13*A*⋯O2^i^	0.84	1.99	2.808 (2)	164
O13—H13*B*⋯O12^ii^	0.84	2.42	3.238 (2)	163
O14—H14*A*⋯O4^iii^	0.84	2.59	3.132 (2)	123
O14—H14*B*⋯O7^iv^	0.84	1.96	2.800 (2)	173
O14—H14*A*⋯O10*B*^v^	0.84	2.23	3.032 (8)	161
C15—H15⋯O6	0.93	2.42	3.258 (3)	150
C15—H15⋯O5^ii^	0.93	2.56	3.238 (3)	130

Symmetry codes: (i) 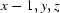; (ii) 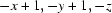; (iii) 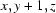; (iv) 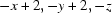; (v) 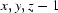.

**Fig. 1.**
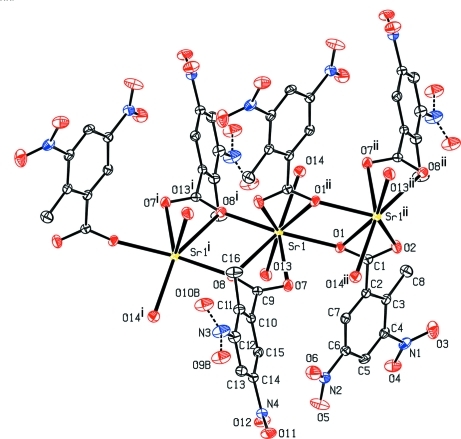
View of the title compound (I). The displacement ellipsoids are drawn at the 30% probability level. H-atoms have been omitted for clarity and only the major component of the disordered NO2 group is represented. [Symmetry codes: (i) -x+1, -y+2, -z; (ii) -x+2, -y+2, -z ]

## supplementary crystallographic information

### Comment

We have reported the synthesiz and crystal structures of Ag(I), Cu(II), trimethyltin(IV) and triphenyltin(IV) complexes of 2-methyl-3,5-dinitrobenzoic acid (Danish, Ghafoor, Ahmad *et al.*, 2011*a*,*b*; Danish, Ghafoor, Tahir *et al.*, 2011; Danish, Tahir *et al.*, 2011). In continuation to synthesize other metal complexes of this ligand, the title compound (I), (Fig. 1) is being reported here.

In the title compound, the Sr^+2^ cation is coordinated to eight O-atoms. Six O-atoms are of four carboxylate groups and two O-atoms from two water molecules. Each ligand of 2-methyl-3,5-dinitrobenzoic acid is chelated and bridged from a single O-atom to the Sr-atoms. In this way infinite one dimensional polymeric chains extend along the [100] direction via four membered planar Sr_2_O_2_ rings [Fig. 1]. The Sr—O bond lengths is in the range of [2.4822 (13)–2.8119 (13) Å] compared to [2.401 (7)–3.064 (7) Å] observed in the related crystal structure of bis(µ2–3,5-dinitrobenzoato)-bis(3,5-dinitrobenzoato)-bis(triethyleneglycol)-distrontium(ii) dihydrate (Hundal *et al.*, 2004). The O—Sr—O bond angles are in the range of 47.88 (4)–157.98 (4)°. The distance between Sr to Sr atoms is 4.1786 (3) and 4.2224 (3) Å, whereas the distance between O-atoms in these four membered planes is 3.0888 (18) and 3.3135 (18) Å, respectively. The dihedral angle between two consective Sr_2_O_2_ planes is 85.02 (5)°.

There are two 2-methyl-3,5-dinitrobenzoato groups which differ from each other geometrically. In one ligand, the carboxylate A (O1/C1/O2), nitro groups B (O3/N1/O4) and C (O5/N2/O6) are oriented at dihedral angles of 45.95 (11)°, 13.97 (32)° and 31.65 (20)° with the benzene ring D (C2—C7) [r. m. s deviation of 0.0047 Å], respectively. In the other ligand one nitro group is disordered over three set of sites with occupancy ratio of 0.46:0.32:0.22. In this ligand, the carboxylate E (O7/C9/O8) is oriented at dihedral angles of 69.02 (09)° with the benzene ring F (C10—C15) [r. m. s deviation of 0.0052 Å], respectively. The dihedral angle between D/F is 28.95 (6)°.

The molecules are stabilized in the form of two-dimensional polymeric network along the plane (001) due to intra as well as inter-molecular H-bondings of C—H···O and O—H···O types (Table 1).

### Experimental

Anhydrous strontium chloride (1.585 g, 0.01 mol) of was dissolved in 25 ml distilled water in 100 ml round bottom flask. Sodium salt of 3,5-dinitro-*ortho*-toluic acid (4.96 g, 0.02 mol) was dissolved in 15 ml of distilled water and added to the strontium chloride solution drop-wise. After complete addition, the reaction mixture was refluxed for 3 h. The reaction mixture was cooled to room temperature and given activated charcoal treatment and filtered. The filtrate was concentrated and kept for crystallization. Light brown prisms appeared within one week.

Decomposition point was 620 K.

### Refinement

The O-atoms of one nitro group are fully disordered over three set of sites with occupancy ratio of 0.46:0.32:0.22. The occupancy factors were initially refined restraining their sum to be equal to 1. Then, once stabilized, they were fixed. The coordiantes of these disordered O atoms were refined using restraints (similar distance for all N—O bonds) and their anisotropic thermal displacement parameters were restrained to be all equal.

All H atoms attached to C atoms and N atom were fixed geometrically and treated as riding with (C–H = 0.93–0.96 Å) with *U*_iso_(H) = *xU*_eq_(C), where *x* = 1.5 for methyl and *x* = 1.2 for aromatic H-atoms. H atoms of water molecule were located in difference Fourier maps and included in the subsequent refinement using restraints (O-H= 0.85 (1)Å and H···H= 1.40 (2)Å) with U_iso_(H) = 1.5U_eq_(O). In the last cycles of refinement, they were considered as riding on their parent O atoms.

### Crystal data

**Table:**

[Sr(C_8_H_5_N_2_O_6_)_2_(H_2_O)_2_]	*Z* = 2
*M**_r_* = 573.93	*F*(000) = 576
Triclinic, *P*1	*D*_x_ = 1.820 Mg m^−^^3^
Hall symbol: -P 1	Mo *K*α radiation, λ = 0.71073 Å
*a* = 8.0901 (3) Å	Cell parameters from 3510 reflections
*b* = 11.2278 (4) Å	θ = 2.4–25.3°
*c* = 12.1356 (4) Å	µ = 2.66 mm^−^^1^
α = 93.805 (2)°	*T* = 296 K
β = 104.566 (1)°	Prism, light brown
γ = 98.971 (1)°	0.30 × 0.26 × 0.22 mm
*V* = 1047.40 (6) Å^3^	

### Data collection

**Table:**

Bruker KAPPA APEXII CCD diffractometer	3772 independent reflections
Radiation source: fine-focus sealed tube	3510 reflections with *I* > 2σ(*I*)
graphite	*R*_int_ = 0.025
Detector resolution: 8.10 pixels mm^-1^	θ_max_ = 25.3°, θ_min_ = 2.4°
ω scans	*h* = −9→9
Absorption correction: multi-scan (*SADABS*; Bruker, 2005)	*k* = −13→13
*T*_min_ = 0.457, *T*_max_ = 0.555	*l* = −14→14
15464 measured reflections	

### Refinement

**Table:**

Refinement on *F*^2^	Primary atom site location: structure-invariant direct methods
Least-squares matrix: full	Secondary atom site location: difference Fourier map
*R*[*F*^2^ > 2σ(*F*^2^)] = 0.023	Hydrogen site location: inferred from neighbouring sites
*wR*(*F*^2^) = 0.057	H-atom parameters constrained
*S* = 1.08	*w* = 1/[σ^2^(*F*_o_^2^) + (0.0257*P*)^2^ + 0.529*P*] where *P* = (*F*_o_^2^ + 2*F*_c_^2^)/3
3772 reflections	(Δ/σ)_max_ = 0.001
324 parameters	Δρ_max_ = 0.41 e Å^−^^3^
6 restraints	Δρ_min_ = −0.33 e Å^−^^3^

### Special details

**Table:**

Geometry. All esds (except the esd in the dihedral angle between two l.s. planes) are estimated using the full covariance matrix. The cell esds are taken into account individually in the estimation of esds in distances, angles and torsion angles; correlations between esds in cell parameters are only used when they are defined by crystal symmetry. An approximate (isotropic) treatment of cell esds is used for estimating esds involving l.s. planes.
Refinement. Refinement of *F*^2^ against ALL reflections. The weighted *R*-factor *wR* and goodness of fit *S* are based on *F*^2^, conventional *R*-factors *R* are based on *F*, with *F* set to zero for negative *F*^2^. The threshold expression of *F*^2^ > σ(*F*^2^) is used only for calculating *R*-factors(gt) *etc*. and is not relevant to the choice of reflections for refinement. *R*-factors based on *F*^2^ are statistically about twice as large as those based on *F*, and *R*- factors based on ALL data will be even larger.

### Fractional atomic coordinates and isotropic or equivalent isotropic displacement parameters (Å^2^)

**Table:**

	*x*	*y*	*z*	*U*_iso_*/*U*_eq_	Occ. (<1)
Sr1	0.72765 (2)	0.986811 (16)	−0.047948 (15)	0.02298 (7)	
O1	0.96083 (17)	0.87808 (12)	−0.07659 (12)	0.0289 (3)	
O2	1.19523 (18)	0.82697 (13)	−0.11206 (14)	0.0399 (4)	
O3	1.0217 (2)	0.39981 (18)	−0.37222 (17)	0.0590 (5)	
O4	0.7722 (2)	0.30089 (14)	−0.36998 (14)	0.0498 (4)	
O5	0.6074 (3)	0.35628 (17)	−0.02052 (16)	0.0604 (5)	
O6	0.7182 (3)	0.51857 (17)	0.09429 (16)	0.0564 (5)	
O7	0.78838 (19)	0.86007 (14)	0.12407 (13)	0.0366 (4)	
O8	0.54635 (17)	0.92959 (13)	0.11675 (12)	0.0301 (3)	
O11	0.6636 (2)	0.48897 (15)	0.47733 (15)	0.0513 (4)	
O12	0.5324 (2)	0.46369 (14)	0.29680 (16)	0.0502 (4)	
O13	0.5123 (2)	0.80020 (14)	−0.15670 (14)	0.0426 (4)	
H13A	0.4157	0.7935	−0.1412	0.064*	
H13B	0.5229	0.7316	−0.1843	0.064*	
O14	0.85434 (19)	1.13859 (13)	−0.16994 (12)	0.0340 (3)	
H14A	0.8052	1.1270	−0.2404	0.051*	
H14B	0.9622	1.1428	−0.1606	0.051*	
N1	0.8935 (2)	0.38576 (16)	−0.33600 (15)	0.0349 (4)	
N2	0.6966 (2)	0.45654 (17)	0.00406 (16)	0.0351 (4)	
N4	0.6078 (2)	0.52745 (16)	0.38607 (17)	0.0342 (4)	
C1	1.0404 (2)	0.80212 (17)	−0.11064 (16)	0.0241 (4)	
C2	0.9477 (2)	0.67147 (17)	−0.14085 (17)	0.0243 (4)	
C3	0.9620 (2)	0.59680 (18)	−0.23461 (17)	0.0262 (4)	
C4	0.8839 (2)	0.47495 (18)	−0.24354 (17)	0.0273 (4)	
C5	0.7966 (3)	0.42722 (18)	−0.16779 (17)	0.0291 (4)	
H5	0.7468	0.3455	−0.1767	0.035*	
C6	0.7863 (2)	0.50508 (18)	−0.07884 (17)	0.0268 (4)	
C7	0.8586 (2)	0.62640 (18)	−0.06419 (17)	0.0268 (4)	
H7	0.8479	0.6774	−0.0038	0.032*	
C8	1.0495 (3)	0.6486 (2)	−0.32047 (19)	0.0379 (5)	
H8A	1.1671	0.6349	−0.3025	0.057*	
H8B	1.0492	0.7342	−0.3181	0.057*	
H8C	0.9884	0.6098	−0.3958	0.057*	
C9	0.6666 (2)	0.87760 (17)	0.16507 (16)	0.0239 (4)	
C10	0.6641 (2)	0.83052 (18)	0.27888 (17)	0.0247 (4)	
C11	0.6965 (3)	0.91066 (19)	0.37863 (18)	0.0304 (5)	
C12	0.6916 (3)	0.8561 (2)	0.47864 (18)	0.0337 (5)	
C13	0.6623 (3)	0.7325 (2)	0.48350 (18)	0.0326 (5)	
H13	0.6617	0.7000	0.5520	0.039*	
C14	0.6341 (2)	0.65978 (18)	0.38341 (18)	0.0281 (4)	
C15	0.6343 (2)	0.70593 (18)	0.28119 (17)	0.0270 (4)	
H15	0.6146	0.6541	0.2144	0.032*	
C16	0.7417 (4)	1.0448 (2)	0.3741 (2)	0.0480 (6)	
H16A	0.7830	1.0591	0.3077	0.072*	
H16B	0.8307	1.0803	0.4418	0.072*	
H16C	0.6405	1.0808	0.3698	0.072*	
N3	0.7184 (3)	0.92991 (19)	0.58829 (17)	0.0518 (6)	
O9A	0.6735 (16)	0.9065 (10)	0.6628 (9)	0.0593 (9)	0.22
O10A	0.8550 (14)	1.0269 (8)	0.6060 (7)	0.0593 (9)	0.22
O9B	0.7307 (8)	0.8739 (6)	0.6789 (5)	0.0593 (9)	0.46
O10B	0.6867 (14)	1.0348 (8)	0.5829 (6)	0.0593 (9)	0.46
O9C	0.8018 (12)	0.8929 (8)	0.6697 (7)	0.0593 (9)	0.32
O10C	0.652 (2)	1.0180 (12)	0.5977 (9)	0.0593 (9)	0.32

### Atomic displacement parameters (Å^2^)

**Table:**

	*U*^11^	*U*^22^	*U*^33^	*U*^12^	*U*^13^	*U*^23^
Sr1	0.02117 (10)	0.02063 (11)	0.02858 (11)	0.00491 (7)	0.00800 (7)	0.00476 (7)
O1	0.0301 (7)	0.0223 (7)	0.0355 (8)	0.0076 (6)	0.0107 (6)	−0.0019 (6)
O2	0.0277 (8)	0.0321 (8)	0.0597 (10)	−0.0007 (6)	0.0187 (7)	−0.0107 (7)
O3	0.0521 (11)	0.0624 (12)	0.0655 (12)	0.0127 (9)	0.0262 (9)	−0.0192 (10)
O4	0.0681 (12)	0.0284 (9)	0.0440 (10)	−0.0036 (8)	0.0089 (8)	−0.0080 (7)
O5	0.0739 (13)	0.0477 (11)	0.0514 (11)	−0.0193 (10)	0.0176 (9)	0.0145 (9)
O6	0.0797 (13)	0.0508 (11)	0.0498 (11)	0.0098 (10)	0.0390 (10)	0.0028 (9)
O7	0.0364 (8)	0.0469 (9)	0.0377 (9)	0.0188 (7)	0.0198 (7)	0.0183 (7)
O8	0.0282 (7)	0.0308 (8)	0.0333 (8)	0.0094 (6)	0.0072 (6)	0.0105 (6)
O11	0.0637 (11)	0.0433 (10)	0.0517 (11)	0.0151 (8)	0.0149 (9)	0.0286 (9)
O12	0.0640 (11)	0.0281 (9)	0.0529 (11)	0.0046 (8)	0.0082 (9)	0.0029 (8)
O13	0.0382 (8)	0.0328 (9)	0.0571 (10)	−0.0010 (7)	0.0206 (8)	−0.0059 (8)
O14	0.0382 (8)	0.0366 (9)	0.0284 (8)	0.0087 (7)	0.0092 (6)	0.0067 (6)
N1	0.0430 (11)	0.0262 (10)	0.0338 (10)	0.0112 (8)	0.0058 (8)	−0.0021 (8)
N2	0.0359 (10)	0.0349 (11)	0.0375 (11)	0.0088 (8)	0.0115 (8)	0.0120 (9)
N4	0.0339 (9)	0.0293 (10)	0.0440 (12)	0.0083 (8)	0.0152 (9)	0.0125 (9)
C1	0.0272 (10)	0.0231 (10)	0.0218 (10)	0.0050 (8)	0.0061 (8)	0.0017 (8)
C2	0.0224 (9)	0.0222 (10)	0.0277 (10)	0.0058 (8)	0.0050 (8)	0.0007 (8)
C3	0.0240 (9)	0.0247 (11)	0.0295 (11)	0.0072 (8)	0.0053 (8)	0.0005 (8)
C4	0.0278 (10)	0.0235 (11)	0.0287 (11)	0.0079 (8)	0.0034 (8)	−0.0030 (8)
C5	0.0308 (10)	0.0188 (10)	0.0342 (12)	0.0042 (8)	0.0028 (8)	0.0020 (9)
C6	0.0255 (10)	0.0265 (11)	0.0288 (11)	0.0060 (8)	0.0063 (8)	0.0067 (9)
C7	0.0276 (10)	0.0256 (11)	0.0271 (11)	0.0075 (8)	0.0065 (8)	−0.0010 (8)
C8	0.0436 (13)	0.0353 (13)	0.0354 (12)	0.0014 (10)	0.0168 (10)	−0.0023 (10)
C9	0.0256 (10)	0.0204 (10)	0.0253 (10)	0.0026 (8)	0.0065 (8)	0.0036 (8)
C10	0.0222 (9)	0.0282 (11)	0.0264 (10)	0.0069 (8)	0.0083 (8)	0.0081 (8)
C11	0.0343 (11)	0.0283 (11)	0.0286 (11)	0.0052 (9)	0.0080 (9)	0.0054 (9)
C12	0.0412 (12)	0.0342 (12)	0.0247 (11)	0.0065 (9)	0.0078 (9)	0.0015 (9)
C13	0.0365 (11)	0.0359 (12)	0.0286 (11)	0.0091 (9)	0.0108 (9)	0.0107 (9)
C14	0.0274 (10)	0.0252 (11)	0.0344 (12)	0.0065 (8)	0.0101 (8)	0.0098 (9)
C15	0.0284 (10)	0.0283 (11)	0.0262 (10)	0.0073 (8)	0.0092 (8)	0.0043 (9)
C16	0.0699 (17)	0.0294 (13)	0.0404 (14)	−0.0003 (12)	0.0126 (12)	0.0039 (11)
N3	0.0852 (17)	0.0397 (13)	0.0271 (11)	0.0132 (12)	0.0077 (11)	0.0043 (9)
O9A	0.109 (3)	0.045 (2)	0.0305 (13)	0.0171 (19)	0.0261 (15)	0.0079 (12)
O10A	0.109 (3)	0.045 (2)	0.0305 (13)	0.0171 (19)	0.0261 (15)	0.0079 (12)
O9B	0.109 (3)	0.045 (2)	0.0305 (13)	0.0171 (19)	0.0261 (15)	0.0079 (12)
O10B	0.109 (3)	0.045 (2)	0.0305 (13)	0.0171 (19)	0.0261 (15)	0.0079 (12)
O9C	0.109 (3)	0.045 (2)	0.0305 (13)	0.0171 (19)	0.0261 (15)	0.0079 (12)
O10C	0.109 (3)	0.045 (2)	0.0305 (13)	0.0171 (19)	0.0261 (15)	0.0079 (12)

### Geometric parameters (Å, °)

**Table:**

Sr1—O1	2.4822 (13)	C1—Sr1^ii^	3.0054 (19)
Sr1—O8^i^	2.5127 (13)	C2—C7	1.388 (3)
Sr1—O13	2.5504 (15)	C2—C3	1.407 (3)
Sr1—O14	2.5884 (14)	C3—C4	1.400 (3)
Sr1—O7	2.5924 (14)	C3—C8	1.499 (3)
Sr1—O2^ii^	2.6413 (15)	C4—C5	1.380 (3)
Sr1—O1^ii^	2.7431 (14)	C5—C6	1.371 (3)
Sr1—O8	2.8124 (14)	C5—H5	0.9300
Sr1—C1^ii^	3.0054 (19)	C6—C7	1.377 (3)
Sr1—C9	3.0502 (19)	C7—H7	0.9300
Sr1—Sr1^i^	4.1785 (4)	C8—H8A	0.9600
Sr1—Sr1^ii^	4.2225 (4)	C8—H8B	0.9600
O1—C1	1.254 (2)	C8—H8C	0.9600
O1—Sr1^ii^	2.7431 (14)	C9—C10	1.515 (3)
O2—C1	1.245 (2)	C10—C15	1.385 (3)
O2—Sr1^ii^	2.6413 (15)	C10—C11	1.402 (3)
O3—N1	1.217 (2)	C11—C12	1.402 (3)
O4—N1	1.222 (2)	C11—C16	1.502 (3)
O5—N2	1.213 (2)	C12—C13	1.379 (3)
O6—N2	1.216 (3)	C12—N3	1.469 (3)
O7—C9	1.245 (2)	C13—C14	1.366 (3)
O8—C9	1.252 (2)	C13—H13	0.9300
O8—Sr1^i^	2.5127 (13)	C14—C15	1.376 (3)
O11—N4	1.221 (2)	C15—H15	0.9300
O12—N4	1.219 (2)	C16—H16A	0.9600
O13—H13A	0.8429	C16—H16B	0.9600
O13—H13B	0.8442	C16—H16C	0.9600
O14—H14A	0.8390	N3—O9A	1.089 (12)
O14—H14B	0.8448	N3—O9C	1.188 (9)
N1—C4	1.479 (3)	N3—O10C	1.207 (17)
N2—C6	1.467 (3)	N3—O10B	1.246 (11)
N4—C14	1.471 (3)	N3—O9B	1.292 (7)
C1—C2	1.515 (3)	N3—O10A	1.388 (10)
			
O1—Sr1—O8^i^	153.44 (5)	O6—N2—C6	118.01 (18)
O1—Sr1—O13	86.98 (5)	O12—N4—O11	124.47 (19)
O8^i^—Sr1—O13	77.46 (5)	O12—N4—C14	117.73 (18)
O1—Sr1—O14	83.09 (5)	O11—N4—C14	117.80 (19)
O8^i^—Sr1—O14	84.89 (4)	O2—C1—O1	123.18 (18)
O13—Sr1—O14	116.73 (5)	O2—C1—C2	118.70 (17)
O1—Sr1—O7	75.24 (4)	O1—C1—C2	117.87 (17)
O8^i^—Sr1—O7	124.33 (4)	O2—C1—Sr1^ii^	61.19 (10)
O13—Sr1—O7	86.64 (5)	O1—C1—Sr1^ii^	65.88 (10)
O14—Sr1—O7	147.29 (5)	C2—C1—Sr1^ii^	154.25 (12)
O1—Sr1—O2^ii^	119.29 (4)	C7—C2—C3	121.55 (18)
O8^i^—Sr1—O2^ii^	83.09 (5)	C7—C2—C1	115.48 (17)
O13—Sr1—O2^ii^	148.57 (5)	C3—C2—C1	122.75 (17)
O14—Sr1—O2^ii^	85.42 (5)	C4—C3—C2	115.64 (18)
O7—Sr1—O2^ii^	84.22 (5)	C4—C3—C8	123.49 (18)
O1—Sr1—O1^ii^	72.29 (5)	C2—C3—C8	120.81 (18)
O8^i^—Sr1—O1^ii^	124.54 (4)	C5—C4—C3	124.09 (18)
O13—Sr1—O1^ii^	157.99 (5)	C5—C4—N1	114.59 (17)
O14—Sr1—O1^ii^	68.99 (4)	C3—C4—N1	121.30 (18)
O7—Sr1—O1^ii^	81.10 (5)	C6—C5—C4	117.31 (18)
O2^ii^—Sr1—O1^ii^	48.12 (4)	C6—C5—H5	121.3
O1—Sr1—O8	121.98 (4)	C4—C5—H5	121.3
O8^i^—Sr1—O8	76.76 (5)	C5—C6—C7	122.30 (19)
O13—Sr1—O8	80.21 (5)	C5—C6—N2	118.54 (18)
O14—Sr1—O8	151.79 (4)	C7—C6—N2	119.16 (18)
O7—Sr1—O8	47.88 (4)	C6—C7—C2	119.08 (18)
O2^ii^—Sr1—O8	71.38 (4)	C6—C7—H7	120.5
O1^ii^—Sr1—O8	104.24 (4)	C2—C7—H7	120.5
O1—Sr1—C1^ii^	96.93 (5)	C3—C8—H8A	109.5
O8^i^—Sr1—C1^ii^	101.60 (5)	C3—C8—H8B	109.5
O13—Sr1—C1^ii^	171.27 (5)	H8A—C8—H8B	109.5
O14—Sr1—C1^ii^	71.62 (5)	C3—C8—H8C	109.5
O7—Sr1—C1^ii^	86.82 (5)	H8A—C8—H8C	109.5
O2^ii^—Sr1—C1^ii^	24.39 (5)	H8B—C8—H8C	109.5
O1^ii^—Sr1—C1^ii^	24.65 (4)	O7—C9—O8	123.75 (18)
O8—Sr1—C1^ii^	91.11 (5)	O7—C9—C10	117.35 (16)
O1—Sr1—C9	98.77 (5)	O8—C9—C10	118.89 (16)
O8^i^—Sr1—C9	100.97 (5)	O7—C9—Sr1	57.03 (10)
O13—Sr1—C9	84.51 (5)	O8—C9—Sr1	67.18 (10)
O14—Sr1—C9	158.76 (5)	C10—C9—Sr1	171.63 (13)
O7—Sr1—C9	23.76 (5)	C15—C10—C11	121.49 (18)
O2^ii^—Sr1—C9	75.16 (5)	C15—C10—C9	117.58 (17)
O1^ii^—Sr1—C9	91.28 (5)	C11—C10—C9	120.90 (17)
O8—Sr1—C9	24.22 (4)	C12—C11—C10	115.53 (18)
C1^ii^—Sr1—C9	87.17 (5)	C12—C11—C16	124.6 (2)
O1—Sr1—Sr1^i^	153.42 (3)	C10—C11—C16	119.78 (18)
O8^i^—Sr1—Sr1^i^	40.93 (3)	C13—C12—C11	124.2 (2)
O13—Sr1—Sr1^i^	75.81 (4)	C13—C12—N3	114.81 (19)
O14—Sr1—Sr1^i^	122.66 (3)	C11—C12—N3	120.96 (19)
O7—Sr1—Sr1^i^	83.55 (3)	C14—C13—C12	117.11 (19)
O2^ii^—Sr1—Sr1^i^	73.31 (3)	C14—C13—H13	121.4
O1^ii^—Sr1—Sr1^i^	120.43 (3)	C12—C13—H13	121.4
O8—Sr1—Sr1^i^	35.83 (3)	C13—C14—C15	122.37 (19)
C1^ii^—Sr1—Sr1^i^	97.70 (4)	C13—C14—N4	118.58 (18)
C9—Sr1—Sr1^i^	60.04 (4)	C15—C14—N4	119.03 (19)
O1—Sr1—Sr1^ii^	38.23 (3)	C14—C15—C10	119.25 (19)
O8^i^—Sr1—Sr1^ii^	153.40 (3)	C14—C15—H15	120.4
O13—Sr1—Sr1^ii^	124.84 (4)	C10—C15—H15	120.4
O14—Sr1—Sr1^ii^	72.32 (3)	C11—C16—H16A	109.5
O7—Sr1—Sr1^ii^	75.50 (3)	C11—C16—H16B	109.5
O2^ii^—Sr1—Sr1^ii^	81.60 (3)	H16A—C16—H16B	109.5
O1^ii^—Sr1—Sr1^ii^	34.06 (3)	C11—C16—H16C	109.5
O8—Sr1—Sr1^ii^	118.10 (3)	H16A—C16—H16C	109.5
C1^ii^—Sr1—Sr1^ii^	58.70 (4)	H16B—C16—H16C	109.5
C9—Sr1—Sr1^ii^	95.98 (4)	O9A—N3—O9C	55.0 (6)
Sr1^i^—Sr1—Sr1^ii^	148.730 (10)	O9A—N3—O10C	83.7 (7)
C1—O1—Sr1	162.80 (13)	O9C—N3—O10C	121.3 (7)
C1—O1—Sr1^ii^	89.47 (11)	O9A—N3—O10B	100.2 (6)
Sr1—O1—Sr1^ii^	107.71 (5)	O9C—N3—O10B	126.1 (7)
C1—O2—Sr1^ii^	94.42 (12)	O10C—N3—O10B	18.6 (6)
C9—O7—Sr1	99.20 (11)	O9A—N3—O9B	29.3 (6)
C9—O8—Sr1^i^	167.96 (13)	O9C—N3—O9B	28.6 (4)
C9—O8—Sr1	88.59 (11)	O10C—N3—O9B	110.3 (5)
Sr1^i^—O8—Sr1	103.24 (5)	O10B—N3—O9B	123.9 (4)
Sr1—O13—H13A	113.0	O9A—N3—O10A	116.7 (7)
Sr1—O13—H13B	133.9	O9C—N3—O10A	87.0 (6)
H13A—O13—H13B	108.6	O10C—N3—O10A	76.0 (7)
Sr1—O14—H14A	115.0	O10B—N3—O10A	60.8 (6)
Sr1—O14—H14B	113.0	O9B—N3—O10A	110.5 (5)
H14A—O14—H14B	108.7	O9A—N3—C12	129.1 (6)
O3—N1—O4	124.04 (19)	O9C—N3—C12	115.3 (5)
O3—N1—C4	118.53 (18)	O10C—N3—C12	123.2 (6)
O4—N1—C4	117.42 (18)	O10B—N3—C12	116.4 (4)
O5—N2—O6	123.78 (19)	O9B—N3—C12	117.4 (3)
O5—N2—C6	118.20 (19)	O10A—N3—C12	111.8 (4)
			
O2—C1—C2—C7	130.4 (2)	C15—C10—C11—C12	−1.7 (3)
O1—C1—C2—C7	−44.0 (2)	C9—C10—C11—C12	−179.61 (18)
O2—C1—C2—C3	−44.4 (3)	C15—C10—C11—C16	175.3 (2)
O1—C1—C2—C3	141.18 (19)	C9—C10—C11—C16	−2.6 (3)
C7—C2—C3—C4	−1.2 (3)	C10—C11—C12—C13	1.7 (3)
C1—C2—C3—C4	173.29 (17)	C16—C11—C12—C13	−175.1 (2)
C7—C2—C3—C8	176.31 (19)	C10—C11—C12—N3	−178.2 (2)
C1—C2—C3—C8	−9.2 (3)	C16—C11—C12—N3	4.9 (3)
C2—C3—C4—C5	0.3 (3)	C11—C12—C13—C14	−0.9 (3)
C8—C3—C4—C5	−177.2 (2)	N3—C12—C13—C14	179.06 (19)
C2—C3—C4—N1	−178.18 (16)	C12—C13—C14—C15	0.0 (3)
C8—C3—C4—N1	4.4 (3)	C12—C13—C14—N4	178.08 (18)
O3—N1—C4—C5	−147.1 (2)	O12—N4—C14—C13	158.1 (2)
O4—N1—C4—C5	31.7 (3)	O11—N4—C14—C13	−22.2 (3)
O3—N1—C4—C3	31.6 (3)	O12—N4—C14—C15	−23.7 (3)
O4—N1—C4—C3	−149.64 (19)	O11—N4—C14—C15	155.96 (19)
C3—C4—C5—C6	0.2 (3)	C13—C14—C15—C10	0.0 (3)
N1—C4—C5—C6	178.77 (16)	N4—C14—C15—C10	−178.07 (16)
C4—C5—C6—C7	0.2 (3)	C11—C10—C15—C14	0.9 (3)
C4—C5—C6—N2	−179.05 (17)	C9—C10—C15—C14	178.88 (17)
O5—N2—C6—C5	−13.9 (3)	C13—C12—N3—O9A	−25.2 (9)
O6—N2—C6—C5	165.09 (19)	C11—C12—N3—O9A	154.8 (8)
O5—N2—C6—C7	166.86 (19)	C13—C12—N3—O9C	39.2 (5)
O6—N2—C6—C7	−14.2 (3)	C11—C12—N3—O9C	−140.8 (5)
C5—C6—C7—C2	−1.1 (3)	C13—C12—N3—O10C	−136.4 (6)
N2—C6—C7—C2	178.14 (17)	C11—C12—N3—O10C	43.6 (7)
C3—C2—C7—C6	1.6 (3)	C13—C12—N3—O10B	−156.3 (5)
C1—C2—C7—C6	−173.25 (17)	C11—C12—N3—O10B	23.6 (6)
O7—C9—C10—C15	−67.7 (2)	C13—C12—N3—O9B	7.3 (4)
O8—C9—C10—C15	111.1 (2)	C11—C12—N3—O9B	−172.7 (4)
O7—C9—C10—C11	110.3 (2)	C13—C12—N3—O10A	136.5 (5)
O8—C9—C10—C11	−70.9 (2)	C11—C12—N3—O10A	−43.5 (5)

Symmetry codes: (i) −*x*+1, −*y*+2, −*z*; (ii) −*x*+2, −*y*+2, −*z*.

### Hydrogen-bond geometry (Å, °)

**Table:**

*D*—H···*A*	*D*—H	H···*A*	*D*···*A*	*D*—H···*A*
O13—H13A···O2^iii^	0.84	1.99	2.808 (2)	164.
O13—H13B···O12^iv^	0.84	2.42	3.238 (2)	163.
O14—H14A···O4^v^	0.84	2.59	3.132 (2)	123.
O14—H14B···O7^ii^	0.84	1.96	2.800 (2)	173.
O14—H14A···O10B^vi^	0.84	2.23	3.032 (8)	161.
C15—H15···O6	0.93	2.42	3.258 (3)	150.
C15—H15···O5^iv^	0.93	2.56	3.238 (3)	130.

Symmetry codes: (iii) *x*−1, *y*, *z*; (iv) −*x*+1, −*y*+1, −*z*; (v) *x*, *y*+1, *z*; (ii) −*x*+2, −*y*+2, −*z*; (vi) *x*, *y*, *z*−1.
